# A molecular approach to triple-negative breast cancer: targeting the Notch signaling pathway

**DOI:** 10.31744/einstein_journal/2024RW0552

**Published:** 2024-01-29

**Authors:** Isabele Pardo, Pedro Brecheret Fagundes, Rafael Santana de Oliveira, Paulo Vidal Campregher

**Affiliations:** 1 Faculdade Israelita de Ciências da Saúde Albert Einstein Hospital Israelita Albert Einstein São Paulo SP Brazil Faculdade Israelita de Ciências da Saúde Albert Einstein , Hospital Israelita Albert Einstein , São Paulo , SP , Brazil .

**Keywords:** Receptors, Notch1, Triple-negative breast neoplasms, Molecular targeted therapy, Signal transduction, Cell proliferation, MicroRNAs

## Abstract

**Introduction:**

Triple-negative breast cancer is an aggressive subtype of breast cancer characterized by the absence of estrogen receptor, progesterone receptor, and human epidermal growth factor receptor 2 expression. This phenotype renders triple-negative breast cancer cells refractory to conventional therapies, resulting in poor clinical outcomes and an urgent need for novel therapeutic approaches. Recent studies have implicated dysregulation of the Notch receptor signaling pathway in the development and progression of triple-negative breast cancer.

**Objective:**

This study aimed to conduct a comprehensive literature review to identify potential therapeutic targets of the Notch pathway. Our analysis focused on the upstream and downstream components of this pathway to identify potential therapeutic targets.

**Results:**

Modulating the Notch signaling pathway may represent a promising therapeutic strategy to treat triple-negative breast cancer. Several potential therapeutic targets within this pathway are in the early stages of development, including upstream (such as Notch ligands) and downstream (including specific molecules involved in triple-negative breast cancer growth). These targets represent potential avenues for therapeutic intervention in triple-negative breast cancer.

**Comments:**

Additional research specifically addressing issues related to toxicity and improving drug delivery methods is critical for the successful translation of these potential therapeutic targets into effective treatments for patients with triple-negative breast cancer.

## INTRODUCTION

Breast cancer is the most common type of cancer in women worldwide and accounted for approximately 15.5% of all cancer deaths in females in 2020. ^( 1 )^ Triple-negative breast cancer (TNBC) constitutes 15-20% of all breast malignancies and is associated with a poor prognosis. ^( 2 )^ It is characterized by the absence of estrogen receptor (ER), progesterone receptor (PR), and human epidermal growth factor 2 (HER2) gene expression. ^( 3 - 5 )^ It exhibits molecular heterogeneity ^( 6 , 7 )^ with six distinct gene expression profile subtypes: basal-like 1 (BL1), basal-like 2 (BL2), mesenchymal (M), mesenchymal stem-like (MSL), immunomodulatory (IM), and luminal androgen receptor (LAR). ^( 7 )^ Both BL-TNBC strains exhibit deficiencies in gene expression associated with DNA repair. Conversely, subtypes M and MSL are characterized by significant expression of genes essential for epithelial-mesenchymal transition (EMT). Additionally, the LAR subtype expresses genes related to certain intracellular signaling pathways mediated by the androgen receptor. ^( 7 )^ Due to limited therapeutic options for TNBC, novel therapeutic approaches are required.

Recent research suggests that the Notch signaling pathway plays an important role in TNBC’s aggressive clinical course and metastatic tendency of TNBC. This pathway is considered fundamental for tumorigenesis and drug resistance in this disease; ^( 8 )^ Notch signaling is an evolutionarily conserved intracellular signaling pathway that regulates cell proliferation, differentiation, and growth. ^( 9 - 11 )^ The transmembrane Notch receptors (NRs) ^( 1 - 4 )^ bind to Notch ligands via cell-to-cell contact. Subsequently, the receptor is cleaved by ADAM and processed by gamma secretase, which forms the Notch Intracellular Domain (NICD) that enters the nucleus and forms a transcriptional activation complex involved in the activity of many genes. ^( 12 , 13 )^ However, several downstream elements of the Notch pathway remain unknown.

This study aimed to outline molecular targets within Notch- and Notch-associated pathways to aid in the future development of TNBC therapy.

### Notch inhibition pathways

**Figure 1:**
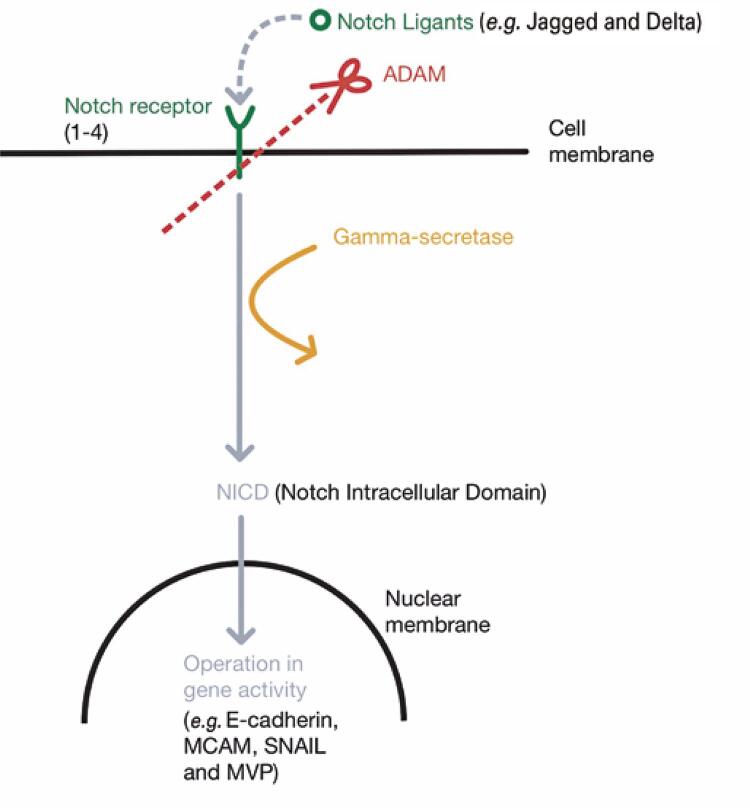
Simplified Notch pathway

Source: adapted from Sprinzak D, Blacklow SC. Biophysics of notch signaling. Annu Rev Biophys. 2021;50(1):157-89. ^( 12 )^

The Notch signaling pathway ( Figure 1 ) is initiated by the activation of ligands that bind to the NR. The NR consists of an intracellular portion, an extracellular negative regulatory region, and a long extracellular tail that connects to the DSL (Delta/Serrate/lag-2) ligand present in an adjacent cell. ^( 14 )^ The interaction generates a biomechanical force on Notch protein that “stretches” it to expose the negative regulatory region (NRR). Subsequently, ADAM protein (ADAM 7 and ADAM10) in the extracellular space cleave NRR, forming a zymogen. ^( 14 )^ The membrane protein gamma-secretase then processes the zymogen to produce the NICD, This complex is directed to the cell nucleus and couples with the RBPJ complex to form the Notch Transcriptional Activation Complex (NTC), which regulates the transcription of a variety of genes. ^( 15 )^

Hence, this pathway has three main possible targets: Notch ligands and activators (such as Jagged, Delta, and gamma-secretase), Notch receptor proteins (including Notch 1-4; NICD), and downstream proteins (such as melanoma cell adhesion molecule (MCAM); major vault protein (MVP)). In addition, the strategy to regulate its function varies since the Notch receptor is highly active in the cell and interacts with other signaling pathways. ^( 16 )^ These different methods are explained in further detail in this section.

The image describes the activation process of Notch receptors: once bound to Notch-ligands (such as Jagged and Delta), the resulting structure is cleaved by ADAM protein and processed by gamma-secretase. This results in the formation of NICD, which enters the nucleus and acts as a gene activity regulator. ^( 12 )^

### Inhibiting Gamma-secretase to downregulate the Notch signaling pathway

Gamma-secretase inhibitors (GSI) are among the most well-studied Notch pathway inhibitors. However, this potential therapy has not yet reached clinical approval despite promising preclinical results. ^( 17 )^ Gamma-secretase inhibitors combined with doxorubicin promote apoptosis and stop the cell cycle *in vitro* , suggesting that GSI enhances the anti-tumor activity of doxorubicin in MDA-MB-231 cells. ^( 18 )^ In addition, GSI and doxorubicin alone partially impede tumor growth without significant side effects *in vivo* . ^( 18 )^ This synergistic antitumor efficacy though combination therapy results in significant weight loss in mice. ^( 18 )^

Some clinical trials using other GSIs to treat breast cancers show limited efficacy as standalone therapies; however, combined therapy has shown promise. ^( 19 )^ Specific treatment of TNBC involving a phase Ib study using a GSI (PF-03084014) combined with docetaxel shows moderate efficacy in patients. ^( 20 )^ Although overall success is still lacking, a better understanding of combination therapy may lead to better outcomes.

### Downregulating the Notch pathway by targeting HIF1α or XBP1 proteins

The Notch pathway is regulated by various proteins, including hypoxia-inducible factor 1α (HIF1α). Recent studies show that overexpression of this protein is associated with poor prognosis in patients and lung metastasis in a mouse model. ^( 21 )^ Therefore, deleting HIF1α reduces primary tumor growth, lung metastasis, and increases overall survival. ^( 22 )^ These findings indicate that HIF1α is a possible therapeutic target.

XBP1 plays an important role in tumorigenicity (especially in TNBC) by regulating HIF1α targets. ^( 21 )^ Therefore, XBP1 may be another target for TNBC treatment since its inhibition results in the deceleration of tumor growth *in vitro* . ^( 23 )^

### Downregulating the Notch pathway via USP9X and TRB3

Pseudokinase tribble homolog 3 (TRB3) is upregulated during periods of stress, such as in tumor microenvironments. ^( 24 )^ It forms a multiprotein complex with ubiquitin-protein ligase mind bomb 1 (MIB1) and deubiquitinase USP9X, which protects the first two from degradation. ^( 25 )^ This complex stimulates the Notch signaling pathway by facilitating JAG1 ^( 24 )^ and promoting ubiquitination-mediated endocytosis. ^( 26 )^

A preclinical study inhibited SP9X with G9 in a murine TNBC model to demonstrate its effect on reducing pathway activity, production of proinflammatory cytokines, and tumor growth. ^( 25 )^ The work indicates that USP9X is a potential cancer therapy with minimal collateral effects. ^( 25 )^

An *in vitro* study testing the inhibition of TRB3 in MDA MB231 cells using siRNA led to reduced cell proliferation. ^( 24 )^ TRB3 promotes the MAPK/ERK signaling pathway, which regulates JAG1 expression in breast cancer. ^( 27 )^ Finally, a comparison of the presence and absence of the TRB3 gene (upregulated under stress) in healthy mice suggested that the pseudokinase has no effect on their physiological health, ^( 28 )^ demonstrating that TRB3 is a potential target to reduce tumorigenesis with minimal side effects.

### Simulating Notch degradation via DTX3

DCAF13 is a Notch4 pathway activator, which acts through DTX3. ^( 29 )^ It is an RNA binding protein (RBP) upregulated in TNBCs ^( 30 )^ that binds to DTX3 mRNA 3’UTR and reduces its stability. ^( 30 )^ DTX3 induces the ubiquitination and degradation of the Notch4 protein, which regulates mesenchymal-like breast cancer stem cells by stimulating SLUG and GAS1. ^( 31 )^

DCAF13 overexpression promotes the invasion and metastasis of MDA-MB-231 TNBC cells *in vitro* , whereas its knockdown suppresses these TNBC characteristics. ^( 30 )^ Hence, an inhibitory effect on DCAF13 or an enhancing effect on DTX3 may serve as therapeutic targets for TNBC.

### IMR and PRI-724 inhibiting Notch and Wnt/β-catenin signaling pathways

An inhibitor of mastermind recruitment (IMR) affects the Notch transcriptional complex that regulates Notch and impedes tumor growth. ^( 32 )^ There is crosstalk between Notch and Wnt/β-catenin pathways in TNBC, ^( 33 )^ although molecules that block Wnt/β-catenin pathways (including PRI-724) are not currently in use to treat breast cancers. ^( 34 )^ Nevertheless, a study by Nasser et al. ^( 33 )^ tested the synergy between IMR-1 and PRI-724 to treat MDA-MB-231 TNBC cells *in vitro* . The results showed that both inhibitors (either alone or combined) reduced the expression of Hes-1, cyclin D1, and VEGF while increasing the expression of β-catenin protein and caspase-3. ^( 33 )^ These findings suggest a significant crosstalk between Notch and Wnt/β-catenin pathways, highlighting their potential to induce apoptosis and decrease angiogenesis, proliferation, and migration. ^( 33 )^

### Triptonide degradation of Notch and Twist proteins

Triptonide derived from the Chinese medicinal herb *Tripterygium wilfordii* has an inhibitory effect on the oncoproteins Notch1 and Twist1. ^( 35 )^ It causes the degradation of Notch and Twist proteins while maintaining their RNA levels *in vitro* . It also inhibits the NF-κB signaling pathway and reduces the expression of genes involved in tumor metastasis and angiogenesis, such as N-cadherin, VE-cadherin, and vascular endothelial cell growth factor receptor 2 (VEGFR2).

This study also analyzed the anti-tumor and anti-metastatic characteristics of triptonide in xenograft mice injected with TNBC MDA-MB-231 cells into their breasts. The Control Group showed a tumor size of 2400mm ^3^ , whereas triptonide-treated mice exhibited a tumor size of only 100mm ^3^ . ^( 35 )^ Finally, the substance did not cause any obvious complications in the mouse organ index. ^( 35 )^

### Short interfering RNA-mediated silencing of Notch, STAT3, and β-catenin genes

Short interfering RNA (siRNAs) dimerize with complementary RNA to silence genes involved in post-transcriptional regulation at extracellular and intracellular locations. This offers easy synthesis and high selectivity. ^( 36 )^

Additionally, siRNA treatment can be used synergistically with different combinations of siRNAs in TNBC cells. ^( 37 )^ Single and combination siRNA treatments of STAT3 (involved in tumor growth and drug resistance ^( 37 )^ ), Notch1 (related to tumor formation and aggressiveness ^( 38 )^ ), and β-catenin (associated with cancer cells proliferation rate and resistance ^( 39 )^ ) in the MDA-MB-231 TNBC cell line demonstrated that enhanced chemosensitization to doxorubicin and decreased cell viability. ^( 16 )^ This might confirm that silencing genes is a viable strategy to enhance the effectiveness of conventional chemotherapy. However, it is important to consider difficulties in cell delivery, which may lead to unwanted side effects. ^( 16 )^

### MicroRNAs targeting Notch1

An additional approach to regulate Notch signaling involves the use of microRNAs (miRNAs) (especially miR-3178), which dimerize and degrade complementary RNAs. ^( 40 )^ A recent study found that miR-3178 expression is significantly downregulated in TNBC and serves as a prognostic factor. ^( 40 )^ Transfecting this RNA into TNBC cell lines suggests that miR-3178 exerts its antitumor effects by targeting Notch1 expression according to the observed proliferation, migration, and epithelial-to-mesenchymal transition (EMT) suppression. ^( 40 )^ Furthermore, there was a significant decrease in tumor volume in nude mice using this miRNA. ^( 40 )^ miR-3178 upregulation in TNBC cells reduces their mesenchymal characteristics, indicating the potential inhibition of EMT through the regulation of Notch using miR-3178. ^( 40 )^ Hence, this strategy represents a promising treatment for TNBC. However, no studies have focused on the side effects of this treatment.

### Nanoparticle co-delivery of Notch1 antibodies and ABT-737

Bcl-2 is an anti-apoptotic protein overexpressed in TNBC. ^( 41 )^ Bcl-2 binds to Bax and inhibits the release of cytochrome C from the mitochondria, thus preventing apoptosis. ^( 42 , 43 )^ ABT-737 is an inhibitor of Bcl-2; however, its use is limited owing to its poor bioavailability and association with thrombocytopenia. ^( 44 , 45 )^

Valcourt et al. ^( 46 )^ reported the synergistic coencapsulation of ABT-737 and Notch1 inhibitors in poly(lactic-co-glycolic acid) nanoparticles (N1-ABT-NPs). This approach regulates Notch and Bcl-2 signaling, while upregulating Noxa (a pro-apoptotic protein) to suppress cell viability and proliferation. Moreover, N1-ABT-NPs exhibit preferential accumulation in TNBC tissues compared to non-cancerous tissues and demonstrate a reduction in the tumor burden in mice. However, there is unintended accumulation outside the target region of interest, such as in the liver. ^( 46 )^ Hence, further developments (including dose adjustment and improvements in encapsulation techniques) are required to optimize its efficacy and minimize potential side effects.

### SiRNA-mediated Syndecan-1 reduction

Syndecan-1 is a surface heparan sulfate proteoglycan co-receptor of multiple biological factors, such as growth factors. There is a positive correlation between Syndecan-1, CD44 (a CSC protein marker), and Notch1 transcription in triple-negative inflammatory breast cancer (IBC) human tissue samples, whereas this correlation is absent in non-IBC samples. ^( 47 )^ Furthermore, siRNA-mediated Syndecan-1 depletion reduces the CD44(+) CD24(-) by 19.5% in SUM-149 and SKBR3 cells compared to the control. ^( 47 )^

Syndecan-1 downregulation reduces tumor angiogenesis, 3D spheroid formation, and colony formation in TNBC cells (SUM-149, MDA-MB-468, and MDA-MB-231). ^( 48 )^ However, further research is necessary to explore the potential side effects.

### Major vault protein inhibition to reduce cisplatin resistance

Major vault protein is overexpressed in TNBC cells and appears to be regulated by Notch1. ^( 49 )^ Both molecules are linked to cisplatin resistance and EMT progression, making them potential targets for TNBC treatment.

Notch1 knockdown in MDA-MB-231 cells downregulates MVP, reduces cisplatin resistance, and reverses EMT. ^( 49 )^ This provides evidence that inhibiting Notch or MVP could increase the efficacy of cisplatin as a treatment for TNBC. ^( 49 )^

### Melanoma cell adhesion molecule inhibition to increase chemotherapy sensitivity

Melanoma cell adhesion molecule is a product of the Notch signaling pathway that acts as an activator of EMT in breast cancer. ^( 50 )^ The Notch1/MCAM axis enables self-renewal ^( 51 )^ and upregulates classic chemoresistant proteins such as P-gp and MRP1. ^( 52 )^

The knockdown of Notch1 in MDA-MB-231 cells increases their sensitivity to cisplatin via a positive correlation with MCAM TNBC. ^( 52 )^ In murine models, tumor proliferation and weight were reduced in the Notch-inhibited group compared to those in the Control Group without any documented side effects. ^( 52 )^ This provides evidence that Notch inhibitors or MCAM monoclonal antibodies can amplify chemotherapy efficacy in patients, although further studies are required.

## COMMENTS

In conclusion, treatment options for triple-negative breast cancer are limited owing to a lack of progesterone and estrogen receptors and human epidermal growth factor 2 expression. ^( 3 , 4 )^ Currently, treatments such as Notch ligands (Jagged and Delta) and Notch receptors can target the upstream part of the pathway. Furthermore, other reactions maintain the signal once the extracellular receptor is stimulated, such as gamma secretase, which can be targeted. Finally, it is possible to target downstream molecules, including specific products that help triple-negative breast cancer growth (including E-cadherin, melanoma cell adhesion molecule, and Snail). For these treatments, siRNAs and miRNAs can be used to reduce the translation of targets or antibodies that destroy proteins.

There are several potential therapeutic targets of the Notch pathway. Although promising, they are mostly in their initial development. In addition, many aspects of this pathway are not fully understood. Therefore, additional research focusing on toxicity and proper drug delivery is required.
